# Dimensions of gender relations and reproductive health inequity perceived by female undergraduate students in the Mekong Delta of Vietnam: a qualitative exploration

**DOI:** 10.1186/1475-9276-11-63

**Published:** 2012-10-24

**Authors:** Thanh Cong Bui, Christine M Markham, Michael W Ross, Mark L Williams, R Palmer Beasley, Ly TH Tran, Huong TH Nguyen, Thach Ngoc Le

**Affiliations:** 1 Division of Health Promotion and Behavioral Sciences, School of Public Health, The University of Texas - Health Science Center at Houston, 7000 Fannin Street, UCT 2610C, Houston, TX, USA; 2 Department of Health Policy and Management, Robert Stempel College of Public Health and Social Work, Florida International University, Florida, USA; 3 Division of Epidemiology, Human Genetics & Environmental Sciences, School of Public Health, The University of Texas - Health Science Center at Houston, Houston, TX, USA; 4 Pham Ngoc Thach University of Medicine, Ho Chi Minh City, Vietnam; 5 Department of Sociology, School of Social Sciences and Humanities, Can Tho University, Can Tho City, Vietnam

**Keywords:** Gender relation, Gender equity, Women’s health, Reproductive health, Vietnam, Undergraduate student.

## Abstract

**Introduction:**

Increasing evidence indicates that gender equity has a significant influence on women’s health; yet few culturally specific indicators of gender relations exist which are applicable to health. This study explores dimensions of gender relations perceived by female undergraduate students in southern Vietnamese culture, and qualitatively examines how this perceived gender inequity may influence females’ sexual or reproductive health.

**Methods:**

Sixty-two female undergraduate students from two universities participated in eight focus group discussions to talk about their perspectives regarding national and local gender equity issues.

**Results:**

Although overall gender gaps in the Mekong Delta were perceived to have decreased in comparison to previous times, several specific dimensions of gender relations were emergent in students’ discussions. Perceived dimensions of gender relations were comparable to theoretical structures of the Theory of Gender and Power, and to findings from several reports describing the actual inferiority of women. Allocation of housework and social paid work represented salient dimensions of labor. The most salient dimension of power related to women in positions of authority. Salient dimensions of cathexis related to son preference, women’s vulnerability to blame or criticism, and double standards or expectations. Findings also suggested that gender inequity potentially influenced women’s sexual and reproductive health as regards to health information seeking, gynecological care access, contraceptive use responsibility, and child bearing.

**Conclusion:**

Further investigations of the associations between gender relations and different women’s sexual and reproductive health outcomes in this region are needed. It may be important to address gender relations as a distal determinant in health interventions in order to promote gender-based equity in sexual and reproductive health.

## Introduction

The goal of gender equality has been a national agenda item in Vietnam since 1946 [1], and strides have been made over the past two decades [1-4]. Noticeable examples are women’s participation in the paid labor force, reduced gender pay gap, and equal opportunity for education [1,2,5]. Despite progress, a recent national survey and international reports show that some aspects of Vietnamese men’s and women’s relations are still far from equal or equitable [6,7]. Sociocultural systems and doctrine, such as patriarchal kinship, have barely changed. Women are still primarily responsible for household chores and caring for family members. Major household expenditures remain largely under men’s control and authority. Men are often regarded as the family bread-winner. Women are expected to be obedient, submissive and self-sacrificing in order to maintain family harmony [1,8]. Contemporary versus traditional role expectations and norms for women in the changing Vietnam society have resulted in unresolved contradictions [8]. For example, in order to advance economically through social paid work and to fulfill traditional family responsibilities, women must work longer hours than men and have comparably little time for rest or leisure [8,9].

Similar to the national picture of gender relations, women in the Mekong Delta region have historically been viewed as inferior to men. Son preference, for example, persists, as it does in the country’s other seven regions [2,7]. Also, the difference between male and female literacy in the Mekong Delta is the third highest among Vietnamese regions that document literacy levels by gender [2]. Nevertheless, there is evidence that some current dimensions of gender relations in the Mekong Delta are distinct from other regions. For example, the perception that women are capable of holding positions in production or business is less common in the Mekong Delta (4.2% agreed) than in the Red River Delta (11.6%) [7]. In contrast, the Mekong Delta has the highest rates of unregistered marriage (39.5%) and divorce (4%), of which more than 50% are initiated by the wives. These figures suggest that women in the Mekong Delta might have gained more autonomy in their marriages [7].

It is widely accepted that gender equity has a significant influence on many crucial areas including women’s health. Given the increased trend of mainstreaming gender issues into health programs, there is a growing body of literature to understand gender-relation dimensions, which are sensitive and specific for different populations and which are applicable to health [10-15]. Nonetheless, capturing and measuring relevant indicators of gender equity or women’s empowerment have been challenging [11,16-18]. Many international organizations working in Vietnam, as well as Vietnamese committees, have developed large-scale, gender-sensitive indicators [1,19-22] in order to monitor and assess programs or policies. However, these macro socioeconomic perspectives do not necessarily facilitate understanding of young women’s own ways of seeing and internalizing gender relations and power. Moser (2007) writes that items from this source of measures have often been driven by political forces and fundable priorities rather than by beneficiaries’ viewpoints [21]. There are very few credible peer-reviewed studies applicable to Vietnam that explore women’s self-perceptions regarding gender relations. Santillan’s work to develop indicators to assess women’s empowerment partially filled this measurement gap [12]. However, this study was designed to measure women’s empowerment in sexual and reproductive health rather than gender relations, and it was conducted in two northern provinces of Vietnam. Studies on gender matters in general in the south and southwest of Vietnam are scarce [7,23]. Additionally, studies by Vietnamese authors are often atheoretical [23], which may explain the paucity of domains measured. Finally, most of these studies employed households as the research unit within married populations. Gender relations in other institutions, such as schools, workplaces, media, or markets, remain underexplored.

As a pilot study for our research on gender relations and young women’s ability to pursue sexual and reproductive health, this study aims to discover salient *perceived* dimensions of gender relations in the Mekong Delta culture. Findings from this study will suggest potential psychological quantitative measures or indicators of gender relations in future health equity research and projects; hence, they will contribute to strengthening theoretical application and measurement in Vietnam. In addition, the study qualitatively examines how this perceived gender inequity may influence female students’ sexual or reproductive health. Although many health researchers are beginning to recognize the importance of gender relations, to date relatively few empirical health research studies have explicitly and purposefully incorporated a gender relations framework [14]. Thus, study findings may contribute to this growing body of literature and to the diverse perspectives pertaining to gender relations and health.

## Method

### Procedure

In March 2009, we conducted four focus group discussions with 32 students in Can Tho University and four groups with 30 students in An Giang University - the two largest universities in the Mekong Delta. Each group consisted of 6–8 participants. Since this pilot study aimed to explore possible diverse dimensions of gender relations, we targeted participants who might be expected to hold differing viewpoints on salient dimensions. Specifically, based on the two main theoretical structures of gender relations – labor and power [24] – we purposively selected students who studied different majors (especially with regard to the division of ‘men’s majors’ versus ‘women’s majors’) and who were at different positions of authority in class or school (e.g., class captain, Youth Union president). We also ensured that our sample included students from different provinces in the Mekong Delta. To recruit students, we first consulted with the Youth Union to help select female students who were in various positions in different school associations (e.g., Youth Union at school and class level, hometown fellow associations). Then, we asked these students to refer someone else who was different in one or more of the characteristics listed above.

Prior to conducting focus group discussions, we obtained informed consent from each participant. One subject refused to participate due to a time conflict. Discussions took place in empty classrooms in the university and lasted approximately 90 minutes. To ensure confidentiality, we requested participants use assigned, unique identification numbers instead of their names during the discussion. Additionally, we asked participants not to mention anyone’s name in their stories and not to talk about the discussions outside the room. All group discussions were audio-taped and transcribed verbatim. The facilitator and note takers were female research assistants who had prior experience conducting qualitative studies. We provided an additional training session to the research assistants at each university, and we met with them after each group discussion for feedback and problem solving. The study was approved by both universities’ boards of rectors and by the Institutional Review Board from the University of Texas – Health Science Center at Houston (HSC-SPH-08-0548).

### Measurement

The discussion guide was developed in consultation with our local collaborators, faculty in the field of social sciences and humanity, to ensure the questions’ clarity and sensitivity. The guide was then pilot tested with a group of seven students. The guide as well as quotations in the results section below were translated then back translated between English and Vietnamese to ensure accuracy. The first open-ended question was, ‘In our society, in what aspects do you think men and women are equitable or inequitable? Based on which standards/indicators would you say so?’ We then asked the same question at the institutional level (university) and the interpersonal level (between dating students). Deliberately, we did not define or distinguish ‘equality’ and ‘equity’ in the discussions. We used a common Vietnamese term '*bình đẳng giới*' and let participants determine its meaning for themselves. When one participant mentioned a dimension which resulted in agreement or disagreement from other group members, we recorded the number of participants who (dis)agreed.

### Data analysis

We grounded our qualitative data analysis in the Theory of Gender and Power (TGP), developed by Robert Connell in 1987 [24]. The TGP explains gender relations within three critical structures: the sexual division of labor, the sexual division of power, and cathexis. The division of labor includes social rules that allocate particular types of work to one gender, such as unpaid housework and childcare to women. It also involves gender differentiation in training, employment, exchange, and promotion. In a wider context, it deals with the organization of work like differentiating men’s jobs from women’s jobs, e.g., those with high demand/low control work environments. This division of labor also recognizes women’s preferences for docile and lower positions. The structure of power comprises authority, hierarchies, coercion and control over resources at different levels: national, organizational, familial, and interpersonal. This structure also recognizes such cultural power as patriarchal order and cultural classification of women as weak. The third structure depicts social sexual relationships between people. This structure manifests in the form of social sexualization, prohibitions and incitements, beliefs, standards, and expectations. These shape people’s perceptions about what is ideal, normative, and fair. Conforming to these structures subsequently makes a woman sexually desirable, appealing and socially accepted. These structures are not independent of each other but rather integrate and interweave to explain the social dynamic of gender. Connell proposes these three structures at two different levels: the society as a whole and particular institutions, like families, workplace, and the street. We selected the TGP as a framework for our analysis because it characterizes gender relations with fundamental yet comprehensive and straightforward theoretical structures (i.e. divisions). Although Connell’s early formulation in TGP has been progressively developed and its classical structure as well as some specific features were suggested to be reframed [25,26], we chose to employ the original simplified model for our analysis. This was because we were specifically looking for simple *individually perceived* dimensions of gender relations instead of a complex macro gender hierarchy, and because we were seeking an established directly applicable, operationalizable, and potentially measurable framework to facilitate our future understanding of gender relations on health outcomes.

To analyze qualitative data, we used content analysis techniques with the aid of the ATLAS.ti 5.0 software package (Scientific Software Development GmbH, Berlin, 2008). Using a deductive approach, two researchers independently performed coding the data based on dimensions described in the TGP. Then, we compared and contrasted codes for inter-rater reliability. Less than 10% of the codes varied; these discrepancies were resolved after a discussion between the raters. Next, we collectively compared and contrasted codes against the TGP’s dimensions and structures. Based on the theory, we also used axial and selective coding to revise the original codes (i.e., dimensions) and to generate supercodes (i.e., structures). Consistencies as well as deviances between our empirical data and theoretical explanations were reflected through our systematically identifying codes’ properties, mainly including the frequencies of emerging dimensions, the level of participants’ agreement upon the direction of a certain dimension (equitable vs. inequitable), and the extent to which a dimension was linked to other codes or supercodes. For all quotations below, texts in square brackets indicate our additions to clarify meaning. When we use ‘group’ instead of number of participants, it indicates that all group members agreed upon a discussed point.

## Results

Fifty eight (90%) out of the 62 female students who participated in the eight group discussions were in their third year. Participants came from six different Mekong Delta provinces and were studying some 26 majors. The ratios of those who had a position in class or school versus no position were about 1:1. All participants were of the country’s major Kinh ethnicity, except one who was of the Muong ethnic group. Eighty percent reported no religion, 10% were Buddhist, and the rest were Catholic and *Thiền Lâm* (a school of Zen Buddhism in Vietnam). None was married.

### What characterizes gender relations in students’ perceptions?

#### The division of labor

The most prominent dimensions related to this structure included housework organization, employment opportunities, segregating men’s fields and women’s fields, and the possibility to pursue higher education. Although six students in five groups acknowledged that today men helped with housework and women went beyond ‘kitchen duties’ to join social activities, the unequal allocation of housework and childcare was identified by 23 participants across eight groups.

Women can also get a [paying] job in society, but one constraint is that women must do all housework, by default, often after working hours. Although we now benefit from technology, such as washing machines, women still have to take care of their children. Men may help a little bit, but they often take care of social affairs, they just help in part. The main responsibility still falls on women’s shoulders. Because women have to take care of their families, their positions in society are not as high or strong as men’s, and women cannot reach their full potential in society.

Childcare was also regarded as a woman’s ‘heaven-given function’ (*‘thiên chức’*). These social views on housework and childcare imperceptibly became the norm as well as a standard by which to judge a duteous (*‘đảm đang’*) woman. Because of this, three participants clearly stated that they would be willing to accept the housewife role and not look for paid work, even though they were third-year degree students.

All participants in eight groups acknowledged that women today are more equal to men in joining the social labor force, having paying jobs, and contributing to household income. However, four participants in one group provided in-depth critiques for the current promotion of women’s roles in society:

In contests [for the purpose of promoting gender equity, often held by unions in public organizations] it is often asked what women can do… such as whether women are ‘capable of national affairs and duteous at housework’ (*‘giỏi việc nước, đảm việc nhà’*); they are expected to be good at both work in the society and in the family; it adds a burden for women… So, empowering women turns out to reinforce the traditional roles of women and make women more burdened.

In the mass media, they hardly praise a man who is ‘capable of national affairs, duteous at housework,’ but when there is a successful woman, they portray her as a symbolic character. Why don’t they interview some male leaders to see if they are good at housework?

In addition, opportunities to find a job were believed to be biased towards men. Six participants gave examples of local firms that recruited only men for certain positions, without clear or convincing reasons, including electrical engineering, milk production, computational software programming, aquicultural development, and fertilizer marketing. Seven participants believed that men were highly favorable for lead, management or supervisory positions and in such fields as land management, internet administration, or even at-home private tutoring – a popular part-time job for students. The most frequent reason for this was the perceived difference in competence between men and women (12 participants), men being more capable of leading, communicating, and having a sense of propriety. Another reason for recruiting men was that ‘women will get married after 1–2 years of working and then they must take care of house, which consumes a lot of their time thus they cannot fulfill their duties at work.’

Men’s and women’s fields were also segregated in some academic majors. Although four groups agreed that male and female students had equal opportunities to select study majors in school, the actual selection of certain majors was clearly associated with gender. Examples of men’s fields were construction, mechanics, information technology, electrical engineering, electronics, physical education, land management and rural development. Women tended to major in accounting and preschool pedagogics. ‘A male student who selected preschool pedagogics would be seen as extraordinarily brave.’

I think there is no equality in academic majors. I was admitted to economics and land administration. All of my family elders asked me which one I would study. They advised me to study economics. They said for land administration, girls were weak so could not learn anything [laugh].

With regard to educational opportunities, 14 participants confirmed such equalities as females were able to go to school, to freely select desired majors, to study in non-discriminatory environments, and to perform in academic settings as well as males do. Nonetheless, seven participants referred to the uneven openings for higher education.

Now and then, here and there, people keep saying ‘what’s the point for girls to study too much… later on, they all will get married, they can do nothing [laughing].’ People just think it is over when a female gets married [laughing].

If a female gets a higher education, such as a master’s or doctoral degree, then people often say that it’s difficult for her to get married [laughing] because it is often said that we should be lower than men; men don’t like women who are one-level higher than they due to their pride and patriarchal thinking.

Regarding other aspects of the division of labor, two participants were concerned about the fact that husbands’ families compelled married women to stop working in order to take care of the family. One participant raised a critical question about a labor policy: ‘Why is the mandatory retirement age for women earlier [five years] than that of men while women are still healthy and are capable to work?’

#### The division of power

The situation of women in positions of authority was the most revealing dimension at the societal and institutional levels. Five participants thought that women, just like men, could enter government, could be leaders in governmental or organizational authority bodies, could engage in politics, and could rise to high positions. In contrast, eight participants cited nuances of the imbalances on this dimension. Although there were women in authority positions, their numbers were fewer than men. Usually, men were first-in-command and women were their deputies or assistants. Sometimes, women were put in a board of director position ‘merely in order to have males and females.’

I read a review regarding employees’ competence in an organization. It asked why their leading body consists of only males in the time of [gender] equity. So, people in that organization thought that they should make some upper positions available for females so that outside people would see equity when they looked at the organization. However, the selected female was not really competent. I think this was not good and was unfair. Let’s promote a woman because of her actual competence. Don’t treat women like that because it is intrinsically inequitable; not [be concerned about] how it looks in the eyes of outsiders.

These for-and-against perceptions were similarly observed in discussions at the institutional level. Twelve students perceived a balance in female students’ authority while 16 students perceived an imbalance. ‘Many members of the [school] associations, even a senior female on the executive board, declare that the head position should be reserved for males; it cannot pass to females.’ Another participant said ‘the head teacher of my class used to tell us that male student should be the class captain because females were not good at diplomatic issues.’

One reason that men were more accepted in leadership positions was the belief that they were more skillful in social propriety, which meant that men knew how best to behave in social settings or matters in order to establish good relationships. This was inter-related with a dimension in social cathexis – a double standard on receiving guests and drinking. In the Mekong Delta, meetings are often followed by a reception with alcoholic drinks. While excessive drinking was acceptable for men, a drunk female would be criticized by others or scolded by her parents (three groups agreed).

Another shared dimension between the power structure and cathexis was discussing important matters and making decisions within the family. Three participants argued that the head of a household could be a woman and that decisions were reached with family members’ consent. Five students believed that men were still considered the bread-winners regardless of women’s contribution to the household income. Seven students perceived that a man is still the ultimate decision maker for family issues, with or without prior discussions among other members. Two students explained that valuable properties such as houses, farming lands, and motorbikes were mostly in men’s names. Two groups agreed that wives commonly had control of family incomes, including their husbands’ earnings. Nonetheless, who made decisions regarding large household expenditures seemed still to be open to debate. Finally, a less recurrent but central dimension was patrilocality, which required a wife to move to their husband’s house upon marriage (two groups). There was little discussion about this because the practice was quickly accepted as fact.

#### Cathexis

Eighteen participants in all eight groups brought up the social phenomenon of son preference as a dimension of gender inequity, and five believed that it was observable even among highly educated couples living in urban areas. Two participants judged that the existence of the monogamy law and the two-child policy helped protect women from such social and familial adversities as of having many children or son expectations. The question of whether a female should take the initiative in expressing her love to a male emerged as a debated dimension for gender equality. Tradition expects women to be passive in meeting men and while dating. Six students believed it is now acceptable for women to take the initiative while eight others did not. ‘When a female chases after a male, she will be mocked [as being] eager for masculine attractiveness.’ ‘Some male students joke that being shown expressions of love by a female is a huge dishonor [for the male].’

Fifteen participants across five groups found it inequitable that females were much more vulnerable to blame and criticism for a variety of issues: having premarital sex, premarital pregnancy, breaking up with boyfriends, being childless, sonless, having a spoilt child, and being a single mother for any reason. All members of one group agreed that such traditional views as ‘a spoilt child is the mother’s fault’ (*‘con hư tại mẹ’*) were persistent. An old saying underscores attitudes toward premarital pregnancy: ‘Having a girl is like having a jar of fish sauce hung at an end of the bed’ (*‘có con gái như hũ mắm treo đầu giường’*), which could break at any time and stink up the whole house (due to the shame of premarital pregnancy). Elders and parents tend to strictly supervise their daughters, for example not allowing them to be out late at night or overnight. Nine participants considered this inequitable.

Women were subject to a number of social double standards. Being a virgin until the wedding night was the most commonly reported (18 participants). ‘Society really attaches much importance to virginity of females; …virginity is everything in a girl’s life.’ If a dating couple had sex and it was discovered, the female would be mocked as ‘being foolishly induced’ or ‘self-indulgent’ while the male would not be criticized. Losing one’s virginity takes women’s power away, and results in maltreatment and violence (four participants).

A male friend told me he would not sincerely commit himself to a relationship with [a female who had lost her virginity], it’s just a transient relationship. He thinks if he can have sex with her, then other males can too; she would accept other men.

The next two connected double standards concerned having friends of the opposite sex (seven participants) and promiscuity (13 participants). A woman’s decency would be questioned if she had many male friends. She is supposed to be monogamous with her boyfriend, but he can flirt or tease other girls. An adulteress was condemned more severely than an adulterer.

A man who’s got women all over the place often proudly flaunts his relationships… If a man has relationships with many women, he will be named *‘đào hoa’*, *‘ong bướm’* (lady-killer). If a woman does [the same], she will be disparaged as *‘lăng nhăng’* (misconduct), *‘lẳng lơ’* (light)… Only beautiful words for men.

Double standards were also noted in breaking up and separation (six participants). If a female took the initiative to say goodbye to her boyfriend, she might be belittled such as ‘bring in the new, throw away the old’ (*‘có mới nới cũ’*), ‘fleetingly hedonic’ (*‘vui qua đường’*), or ‘changing boyfriends as often as changing clothes’ (*‘thay bồ như thay áo’*).

With regards to socially emotional attachments, women in the Mekong Delta appeared to conform to such traditional attitudes as meekness, sacrifice, concession, and sufferance.

I think one traditional ideology for Vietnamese women is sufferance (*‘chịu đựng’*). This has many meanings. Women can suffer hardship in order to bring up their children and to pay for their schooling. In terms of sentiment, women suffer their husbands’ oppression or indiscretions, such as drinking, gambling, or violence, for the good of their children and the whole family. So, the word ‘sufferance’ makes women become unduly resigned to their fate.

### Influence of inequitable gender relations on female sexual and reproductive health

Responding to the open-ended question ‘How may the discussed aspects of gender inequity influence women’s sexual or reproductive health?’, 17 participants across seven groups referred to different examples of the negative influence. First, females had less access to sexual and reproductive health information than males because of double standards and social attitudes towards females. One group agreed that ‘men have more rights to access and learn the information,’ and that ‘females are more likely to be scolded when asking elders about sexual matters.’ Another group felt it was more uncomfortable for females to search for this type of sensitive information on the internet because they might be teased for doing so while it was quite a normal thing for males to do. (In the Mekong Delta, the majority of people use public internet shops to access the internet).

Second, vilification hindered girls and women from obtaining sexual and reproductive healthcare such as gynecological examinations. Two groups agreed upon the fact that many women do not get gynecological examinations partially because it made them look like they had done something wrong (e.g., pregnant out of wedlock) and ‘it was something dark and secret to do.’

If I have a health problem, and I want to go for a [gynecological] examination, I feel ashamed and rather afraid of going there. This is because, for example, if we go there and meet an acquaintance, s/he will sneer at us… Even my Mom says so. I have had [gynecological] problems, and I wanted to have an examination, but my Mom told me that I should talk to her so that she could buy some medicine [self-prescribed] for me rather than going [to a clinic]; people would mock me for it. I don’t really know why it is so, but I dare not [contradict] my Mom. Although sometimes I feel discomfort, I just follow some traditional remedies; I haven’t had an examination yet.

Several additional examples highlighted how gender inequity may influence women’s communication and negotiation ability. Two participants believed that unmarried young females were not capable of communicating and negotiating sexual issues. Two other participants perceived that many women in rural areas had to passively and responsively ‘pamper the husbands’ sexual desire’; thus they could not refuse their husbands’ sexual requests during their menstrual cycle or postpartum period. Three participants mentioned that under the social ideas of son preference or a large family with many children and grandchildren (*‘con đàn cháu đống’*), women were expected to have several pregnancies and quick recoveries after giving birth. They could not resist the husbands’ or mothers-in-law’s wishes regarding the number or the sex of the children. Thus, they did not have much control over the number and timing of their pregnancies. ‘Some females do not want to get married because,’ they joked, ‘we will not be respected after getting married and will be considered a baby machine.’ Two other participants in the group said that they had witnessed domestic violence due to a woman’s attempt to resist her husband wishes.

Other inequalities in sexual and reproductive health included women’s responsibility for contraception (three participants) and men’s lack of responsibility regarding unwanted pregnancies (four participants). ‘Men often resist using condoms; they just force women to use contraceptive pills, to use an intrauterine device. All responsibility is on the woman.’ For unmarried females who were sexually active, this was more difficult because of the stigma of getting, carrying and using a contraceptive. When an unmarried couple had an unwanted pregnancy, ‘the male often told the female to seek an abortion, regardless of long-term consequences on the female’s reproductive capacity.’ Additionally, ‘the female, not the male, was often blamed why she let this happen to her.’ Other less salient influences of gender inequalities on reproductive health included doing heavy housework shortly after child bearing (two participants) due to women’s roles, and early marriage (one participant) in some rural families, at which age the girls’ reproductive organs and functions might not yet be fully developed.

We further explored institutional perspectives regarding safe-sex practices by asking the question: ‘What would teachers or school employees think about a male versus a female student if they know that the student talks about safe-sex practices, has had sex and carries a condom?’ Although opinions regarding talking about sexual issues varied from conventional to open-minded, most participants thought that these were not different for males or females. Regarding premarital sex and carrying a condom, most participants perceived that these looked bad, or even terrible, to faculty and school employees. About one half of the participants thought that disdain was similar for males and females while the other half believed females were more harshly criticized.

## Discussion

Overall, we found that the most cited dimensions of gender relations that were perceived by students and which emerged during the group discussions were comparable to and could be linked to the umbrella structures of TGP. Using our best judgment, we summarized the perceived dimensions of gender relations by arranging them in conjunction with the primary TGP structures (Table 1). In line with Connell’s suggestion that the three structures are interdependent and inseparable, we aimed not to isolate structures or dimensions in the table. In fact, we deemed that a number of dimensions corresponded equally with two underlying structures, so we positioned them in cells that cross-cut rows and columns. For example, we observed a strong linkage between female power and double standards regarding premarital sex and separation, which tied females into an unwanted or violent relationship. This observation aligns with Connell’s argument that double standards – a domain of cathexis – have everything to do with greater power.

**Table 1:** Perceived dimensions of gender relations among female undergraduate students in Mekong Delta

**Structures**	**Division of labor**	**Division of power**	**Cathexis**
Division of labor	-Inequitable organization of housework and childcare		
	-Segregation of ‘men’s jobs’ and ‘women’s jobs’ in academic majors and labor markets		
	-Favor of men over women in job recruitment, particularly in high-power positions		
	-Women’s participation in social activities		
	-Pursuit of higher education		
Division of power	-Opportunities to rise to high or leading positions at institutional level	-Protective effect of national laws or policies (e.g., monogamy, two children)	
	-Stereotypes regarding women’s competence in certain fields or types of work (e.g., management)	-Number of women in positions of authority in governmental and local organizations	
	-Differentiation in retirement ages for men and women	-High rate of women as second-in-command to men	
	-Cultural power: definition of women as ‘the weak gender’	-Control over household income and spending	
		-Makes family decisions	
		-‘Bread-winner’	
		-Ownership of valuable properties	
		-Control over sexual requests and fertility decisions (e.g., number of children, birth intervals).	
Cathexis	-The praise of duteousness	-Patrilocality (reside with husband’s family after marriage)	-Son preference
		-Double standard for being a virgin	-Vulnerability to blames for being childless, sonless, having a spoilt child, being single mother
		-Double standard for premarital sex	
		-Double standard for promiscuity	
		-Double standard for separation and shift to a new relationship	

One observation regarding the sexual division of labor in the Mekong Delta was its strong influence on the extent to which women were constrained from further development opportunities, such as higher education or professional advancement. Housework, for instance, was expected of women, and fulfilling housework was socially considered a virtue of women – duteous. Time spent doing housework, then, rivaled time invested in other practices. Women were viewed less favorably in some paid job placements or higher echelons due to their ‘busy time’ for housework, child bearing and child rearing after marriage.

Key dimensions of the TGP division of power were also observed in participants’ perceptions. Beliefs in patriarchal order and power, which interact with the structure of cathexis, place an emphasis on women to abide by such ideals as meekness, obedience, concession, and sufferance or such responsibilities as keeping family harmony and saving face. However, it was of interest that no student raised the issue of patrilineality, which might denote the normalization or the inconsequentiality of this dimension in students’ perceptions. The fact that women were often second-in-command to men contributes evidence to the continuation of an institutionalized authority hierarchy. The cultural power term *‘phái yếu’*, defining women as the weak gender (despite some students’ attempt to correct it as ‘the beautiful gender’), still applied in that female students perceived themselves to be excluded not only from physically demanding industries (e.g., mechanics, construction) but also from high-tech and seemingly high-stress areas (e.g., land and environmental resource administration, rural development, electronics, and computer sciences).

With respect to the structure of cathexis, multiple dimensions corresponded with primary theoretical domains. Double standards and expectations were examples of social desire for male–female relationships in the form of conjunction between prohibitions and incitements. Being a virgin and being reserved and obedient may represent ways of standardizing feminine appeal during the process of sexualizing women. Some dimensions in this structure potentially generated ambivalence in women’s reproductive health decision-making. Because of son preference, for example, a sonless woman, who may have no preference regarding her child’s gender or who may not want more children, may unconsciously repress her own preferences and express her wish to bear a son to be in accord with her husband’s family or social expectations. To be pregnant with another daughter may instill both feelings of love and disappointment.

Although our goal was to identify students’ perceptions of salient dimensions of gender relations rather than to examine the actual current status of gender equity, we reckoned that participants’ perceptions about gender gaps were close to reality and may mirror the national situation. The participants perceived that today’s women are more empowered compared with women in previous decades, but they are not yet equal to men in many aspects. This perception is corroborated by data from the most recent Global Gender Gap Report. In 2008, Vietnam dropped 25 places in gender gap rankings, from 42 in 2007 to 67 out of 128 countries [6]. The lower rank suggests greater gender disparity. This drop was mostly due to the unequal participation and empowerment of men and women in political and economic sectors. The ratios of women to men in parliament and in ministerial positions were .04 and .35, respectively. The ratio of women serving as legislators, senior officials and managers was .28 in 2008. Although equality in education had been the most notable gender achievement in Vietnam [2], the higher the education level, the lower the ratio of female to male enrollment [1,6]. Some reports still indicate gender inequality in fields of study and paid work, such as the under-representation of females in information technology [1,6,27]. According to data provided by the two universities (unpublished data), the percentages of third-year female students in men’s fields were 25.3% for information technology, 13.4% for engineering, 3% for physical education, and 25% for rural development. In contrast, the percentage of female students in elementary-school pedagogics was 80%. Biased responsibilities and unequal participation in housework still widely appear in several reports [1,7,9,28]. The pressure of being ‘capable of national affairs’ in addition to ‘duteous at housework,’ which one group mentioned, was observable in other regions of Vietnam [8]. Men’s names registered as owners of such capital assets as houses, land, motorbikes, and motorboats were more than 80% [7]. Patrilocality remained a dominant form of living after marriage.

Participants’ perceptions pertaining to the potential influence of inequitable gender relations on women’s sexual and reproductive health are supported by external evidence. Sex before and outside marriage was more socially unacceptable for women than for men [7]. Contraceptive use in response to national fertility regulation, together with its costs and risks, is primarily the woman’s responsibility [29]. Comparably, Santillan’s study (2004) found that traditional gender norms encouraged women’s tolerance of contraceptive responsibilities and side effects and discouraged women with gynecological health problems from seeking treatment [12]. In our subsequent survey of 1181 female students at the two universities, we asked to what extent they agreed with the statement ‘If an unmarried woman obtains a gynecological examination, she will be denigrated?’ On a 7-point scale, 12.2% slightly agreed, 17.5% agreed and 4.5% completely agreed [30].

Besides the influence of inequitable gender relations on female sexual and reproductive health which were perceived and discussed by the participants, other latent links between dimensions of gender-based inequities in all three TGP divisions and certain sexual and reproductive health outcomes have been identified in health literature. For example, although Vietnamese national policies has been generously supporting maternity leave, the entitlements attached to maternity leave might be a disincentive to for-profit employers, and hence prevented women from having a paid job [2]. As another example, the female/male ratio at birth of .93, which put Vietnam in the rank of 112 out of 130 countries, implied the persistence of son preference [6]. The Mekong Delta was among the regions with high rates of families supporting the necessity of having a son [7]. Together with low power in communication and decision-making, patrilineality and son preference might lead women to undergo sex selective abortion, which has been documented in Vietnam [31,32].

In order to enhance young women’s equity and autonomy in attaining sexual and reproductive health, it may be necessary to promote gender equity as a distal determinant. The school could be an important agency of gender-equity socialization. Also, understanding the interacting roles of different institutions of gender relations, such as between the school institution and the media institution, may bring new issues to light and suggest helpful interventions. This may be a topic for future gender-and-health studies or programs.

This study contains some noticeable limitations. Purposive sampling means that our sample does not represent the student body overall. Findings wholly rely on participants’ one-time responses, which serves our purpose of understanding students’ perceptions about gender relations but which may not truly or completely reflect reality. Some students might not have answered honestly when discussing sensitive topics in the schools, despite our assurance of privacy and confidentiality. Although group discussion techniques are useful for examining how ideas are perceived and debated, the group atmosphere might also have restrained participants from revealing their personal viewpoints against dominant perceptions. In spite of our attempts to provide an account of the frequency of specific dimensions, these numbers do not imply dimensions’ significance or magnitude in explaining gender relations. Finally, given the focus group discussion research methodology, causal associations between gender relations and women’s sexual or reproductive health issues should not be inferred or generalized.

## Conclusion

Although overall gender gaps in the Mekong Delta were perceived to have declined, several specific dimensions of gender relations were emerged in students’ discussions. The inferiority of women has seemingly been sustained in several key aspects and in specific nuances of these aspects. The findings also suggested that the inequitable gender relationships may influence women’s sexual and reproductive health through several trajectories. Further exploration, measurement and statistical examination of the associations between gender relations and women’s sexual and reproductive health outcomes in this region are needed. Dimensions of gender relations listed in Table 1 may serve as useful measures or suggest variables for future research on gender equity and health in this population. It may also be necessary to initiate and foster activities to enhance in-school gender relations at the university level and at earlier education levels (e.g. high schools) in order to promote gender-based equity in sexual and reproductive health.

## Abbreviation

TGP: Theory of Gender and Power.

## Competing interests

The authors declare that they have no competing interests.

## Authors’ contributions

TCB and CMM were principal investigators of the study and participated in all research stages and activities. MWR, MLW, RPB provided substantial contributions to the theoretical conceptualization, study design, interview guide and questions, data interpretation, and revising the manuscript critically for important intellectual content. LTT and HTN played key roles in conducting interviews, transcribing, coding, and preliminarily analyzing data. TNL provide intellectual and cultural advice, significantly participated in piloting interview questions in Vietnamese, sampling and recruiting techniques, and interpreting results. All authors read and approved the final manuscript.
